# Influence of Carrier Gas Composition on the Stress of Al_2_O_3_ Coatings Prepared by the Aerosol Deposition Method

**DOI:** 10.3390/ma7085633

**Published:** 2014-08-05

**Authors:** Michael Schubert, Jörg Exner, Ralf Moos

**Affiliations:** Department of Functional Materials, University of Bayreuth, Universitätsstraße 30, Bayreuth 95440, Germany; E-Mails: functional.materials@uni-bayreuth.de (M.S.); functional.materials@uni-bayreuth.de (J.E.)

**Keywords:** aerosol deposition, film stress, Al_2_O_3_, carrier gas, room temperature impact consolidation (RTIC)

## Abstract

Al_2_O_3_ films were prepared by the aerosol deposition method at room temperature using different carrier gas compositions. The layers were deposited on alumina substrates and the film stress of the layer was calculated by measuring the deformation of the substrate. It was shown that the film stress can be halved by using oxygen instead of nitrogen or helium as the carrier gas. The substrates were annealed at different temperature steps to gain information about the temperature dependence of the reduction of the implemented stress. Total relaxation of the stress can already be achieved at 300 °C. The XRD pattern shows crystallite growth and reduction of microstrain while annealing.

## 1. Introduction

In order to obtain dense and hard ceramic coatings or films, ceramic materials have typically to be sintered above 800 °C, alumina even sometimes above 1800 °C [1]. In contrast, the Aerosol Deposition (AD) Method is a coating process to obtain dense ceramic films at room temperature with no need for any sintering process. For that purpose, an aerosol of submicron particles is accelerated by a pressure difference through a nozzle into a deposition chamber. There, the particles impact on a substrate and build a dense layer by fracture and plastic deformation of the particles on the surface of the substrate [2]. This mechanism is called room temperature impact consolidation (RTIC). It was first investigated by Akedo *et al.* [2,3,4]. The basic setup for such a process contains an aerosol generator, a deposition chamber, and a vacuum pump. The vacuum pump builds a rough vacuum inside the chamber and inside the aerosol generator where the raw ceramic powder is placed. A detailed figure of the setup can be found in [2,3,5]. By passing a carrier gas through the powder, an aerosol is formed and particles are transported driven by the pressure drop from the aerosol generator into the chamber. By a slit-nozzle, the aerosol jet is accelerated up to several hundred m/s and ejected on the target, forming a ceramic layer at a deposition rate of several µm/min [2,5].

It is the main feature of the AD method that it does not require any sintering process for building dense ceramic layers with a closely packed structure, high strength, and strong adhesion [6]. Therefore, AD is advantageous for a broad spectrum of temperature sensitive substrate materials, including ceramics [2], metals [7], silicon [8], or even polymers [9]. Materials that have been successfully deposited by this process are non-oxide materials (AlN [10], MgB_2_ [11]) as well as oxide materials (Al_2_O_3_ [12], TiO_2_ [5,13], Y_2_O_3_ [14], Pb(Zr,Ti)O_3_ [15], SrTi_1−x_Fe_x_O_3_ [5]), and compound materials (Al_2_O_3_/PTFE [16], ZnS/Diamond [8]).

The characteristics of this deposition process offer a huge variety of applications, for example in MEMS technology or 3D packaging techniques [16]. Especially for that purpose, it is necessary to have a coating with a very small mechanical stress to protect the deposited film against cracks and delamination. Due to the special deposition process which is characterized by fracturing and solidification of the starting powder, high stresses within the layer are reported, e.g., by Kim *et al.* [16]. There the stress was reduced by adding PTFE to the raw Al_2_O_3_ powder. The present study was aimed at decreasing this residual stress within the layer without mixing of the raw powder with any contaminants or dopants. For that purpose, the influence of the carrier gas composition was investigated and the residual stresses of the deposited layers were measured. 

## 2. Experimental

α-Al_2_O_3_ starting-powder with a particle size of *d*_50_ = 0.5 µm was prepared by wet ball milling with cyclohexane as solvent for four hours followed by the removal of the milling solvent with a rotary evaporator. After drying in a circulating air oven at 120 °C to evaporate solvent residues, it was sieved with a 90 µm screen to break up big agglomerates and finally dried in an electrical furnace at 200 °C in air atmosphere. After this procedure, the *d*_50_ = 0.5 µm remained unchanged with agglomerate size of less than 90 µm.

Table 1 lists the deposition conditions for the Al_2_O_3_ films. The pressure in the deposition chamber was 10 mbar, in the aerosol generator 250 mbar. The composition of the carrier gas was changed from 100% N_2_ by adding O_2_ in 20% steps up to 100% O_2_. In a second test series, it was changed from 100% He by adding O_2_ in 20% steps up to 100% O_2_. The Al_2_O_3_ films were deposited on 150 µm thick alumina substrates at room temperature in order to evaluate the bending of the substrates due to the mechanical stress within the coating and for SEM cross-sections. The stress, σ, in the deposited layer was calculated by Equation (1), described by Stoney in [17]

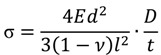
(1)

Herein, *E* stands for the Young’s modulus of the alumina substrate; *d* for the thickness of the alumina substrate; *ν* is the Poisson’s ratio of the alumina substrate; *l* the length of the coating; *D* denotes the measured bending of the substrate; and *t* the thickness of the deposited film.

**Table 1:** Deposition conditions for the Al_2_O_3_ films.

Parameters	Values
Orifice size of the nozzle	10 × 0.5 mm^2^
Distance nozzle—substrate	3 mm
Substrate temperature	Room temperature
Gas flow rate	6 L/min
Carrier gas	O_2_, N_2_, He, and mixtures as indicated in the experiments
Sweep rate	1 mm/s

**Figure 1:**
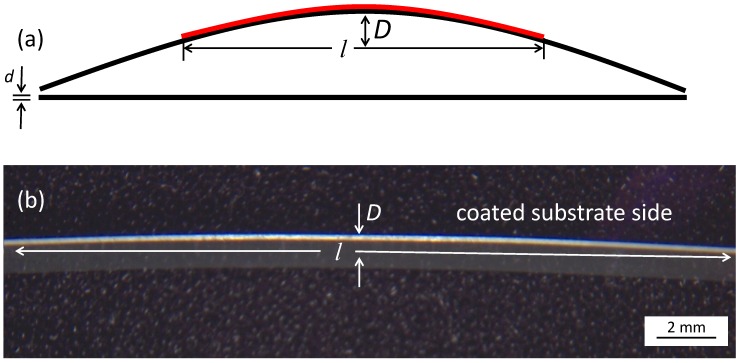
(**a**) Schematic drawing of the flat Al_2_O_3_ substrate without coating and of the bended one after applying the coating (red); (**b**) typical measurement of bending, *D* (235 µm), and length, *l* (19.35 mm), of the layer of a coated substrate. Deposition parameters: 100% He, film thickness *t* ≈ 4 µm.

Figure 1a is a schematic drawing of the alumina substrate. The straight line denotes the alumina substrate before deposition and the bended line shows the behavior after the deposition of an AD film. The bending was analyzed under a light optical microscope (MZM1, Askania, Rathenow, Germany) with the analyzing software Progress Capture Pro (JenOptik, Jena, Germany), cf. Figure 1b. It also illustrates the meaning of the variables of Equation (1).

The microstructural properties of the aerosol deposited films were characterized by scanning electron microscopy (SEM, Leo 1450 VP, Zeiss, Oberkochen, Germany) and high temperature X-Ray diffraction (Bruker D8 ADVANCE, 1200 °C, Karlsruhe, Germany). Therefore, the AD layers were not deposited on alumina substrate but on high temperature stable partially stabilized zirconia (PSZ) substrate, which shows well-defined and distinguishable patterns to avoid interferences with the pattern of the aerosol deposited alumina film. *X'Pert Highscore Plus* software with included algorithms for Rietveld-refinement was used for structural analysis. Crystallite size and microstrain were calculated using the software based on the Rietveld-refinement with pseudo-Voigt fitting function.

The thicknesses, *t*, of the deposited layers were measured by a stylus profilometer (PGK/S2, Mahr, Goettingen, Germany). Post annealing was performed in a chamber furnace in air.

## 3. Results and Discussion

### 3.1. Characterization of the Deposited Films

**Figure 2:**
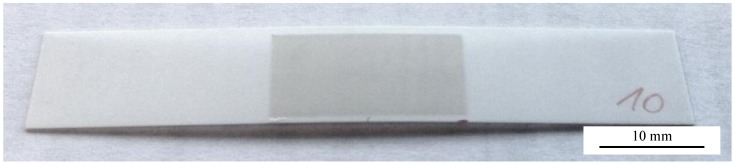
Example for a typical Al_2_O_3_ Aerosol Deposition (AD) layer on an alumina substrate after deposition. Deposition parameters: 80% N_2_/20% O_2_, film thickness *t* ≈ 9 µm.

**Figure 3:**
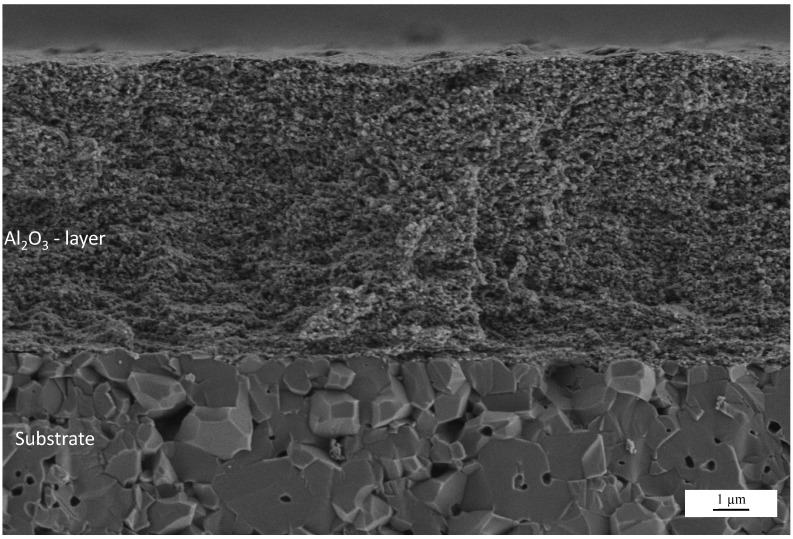
SEM cross-sectional fracture pattern of an Al_2_O_3_ AD layer sprayed on an alumina substrate. Deposition parameters: 100% N_2_, film thickness *t* ≈ 8 µm.

Al_2_O_3_ thick films were successfully deposited on the alumina substrates. A typical result is shown in Figure 2. The grey Al_2_O_3_ AD layer has such a high stress implemented that the substrate bends and the middle of the substrate notably lifts. Cross-sectional SEM images were taken in order to analyze the microstructure of the layer as shown exemplarily in Figure 3.

The difference between the AD layer and the substrate is marked. The Al_2_O_3_ AD-layer is very finely grained whereas the Al_2_O_3_ substrate is coarsely grained with a distinct interface between both. The coating itself has a thickness of approximately 8 µm (as shown in Figure 3 and validated by multiple profilometer measurements in a range of ±0.2 µm) and is homogenous without any porosities or cracks. It appears even denser than the substrate which shows some porosity. The AD layer adheres strongly to the substrate as there is no visible liftoff from the substrate. The adherence is also validated by tape tests. The films also withstand scratching with a metallic pin.

### 3.2. Carrier Gas Influence on Film Stress

In order to calculate the stress within the AD layer, the bending, *D*; the length of the AD layer, *l*; and the thickness of the layer, *t*, were measured. Figure 1b illustrates a coated substrate with the measurement of the bending and the length of the layer. Together with the material parameters of the substrate *E* = 380 GPa, *ν =* 0.23 and *d* = 0.15 mm, the stress of the layer was calculated for layers deposited with different carrier gas mixtures as already described.

**Figure 4:**
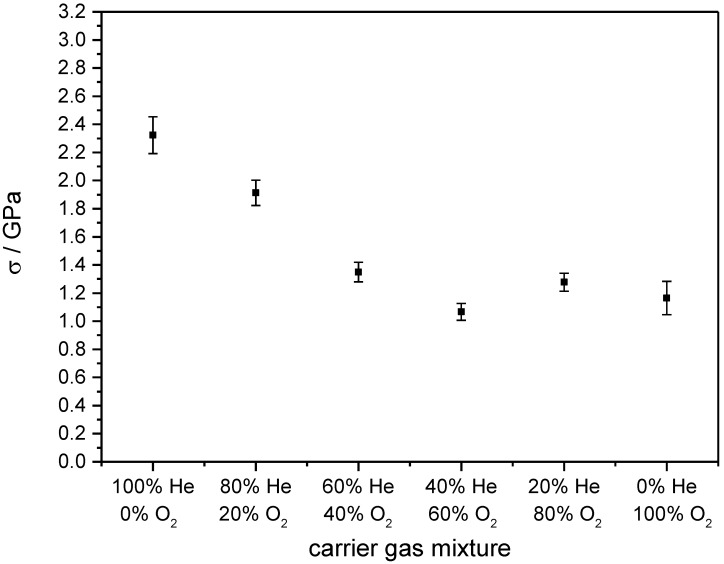
Film stress in dependence of He/O_2_ mixtures as carrier gas. Error bars describe the calculation of errors of the used measurement devices.

**Figure 5:**
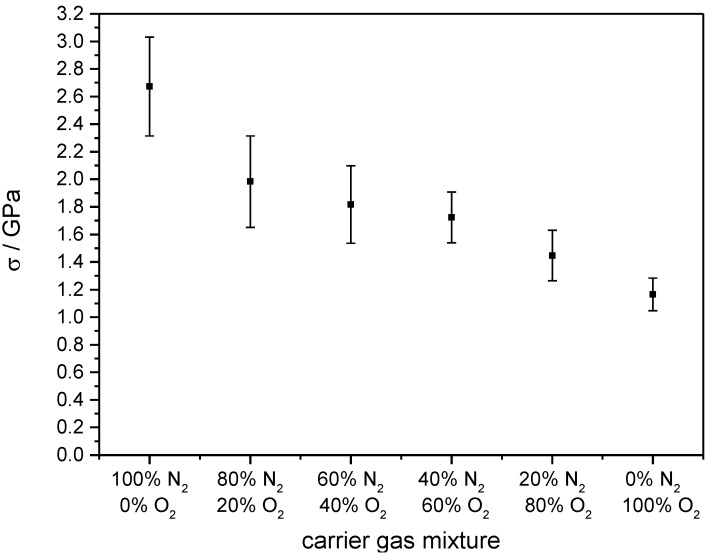
Film stress in dependence of N_2_/O_2_ mixtures as carrier gas. Error bars describe the calculation of errors of the used measurement devices.

Figure 4 elucidates the influence of He/O_2_ ratio in the carrier gas. Starting with 2.3 GPa when sprayed in 100% He, the stress can be halved by adding 40% O_2_ with 60% He as a balance. A further increase of O_2_ does not change the obtained stress anymore. A similar trend shows the mixture of N_2_ and O_2_ as carrier gas (Figure 5). By adding O_2_, the film stress can be reduced successively. Starting with an even higher value than He, pure N_2_-sprayed layers exhibit a stress over 2.6 GPa. By continuously adding O_2_, it can be reduced monotonically down to 1.2 GPa if sprayed in 100% O_2_. The error bars were calculated based on the propagation of uncertainties in Equation (1) with the errors of the used measurement devices.

**Figure 6:**
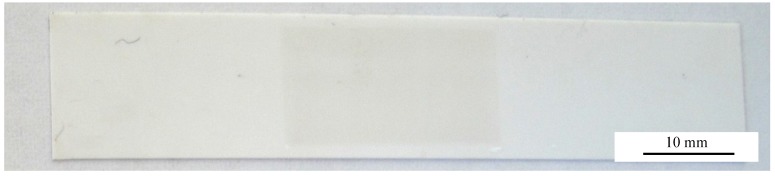
Example for a typical Al_2_O_3_ AD layer on an alumina substrate after deposition. Deposition parameters: 100% O_2_, film thickness *t* ≈ 8 µm.

These results may be explained with the oxidizing atmosphere inside the deposition chamber when using O_2_ as the carrier gas. Here, there stoichiometric composition of Al_2_O_3_ could be reached much more easily than using inert, almost oxygen-free gases like He or N_2_ that could possibly build Al_2_O_3−δ_ non-stoichiometric compositions. Reasons for this could be the deposition mechanisms, for which it is assumed that an unsaturated/reactive surface is built due to the fraction of the particles into nano-sized fragments [2]. The darker color, a hint for oxygen vacancies in alumina, of the 80% N_2_/20% He sprayed layer in Figure 2 compared to the 100% O_2_ sprayed layer in Figure 6 supports this.

### 3.3. Annealing of Coated Al_2_O_3_ Substrates

**Figure 7:**
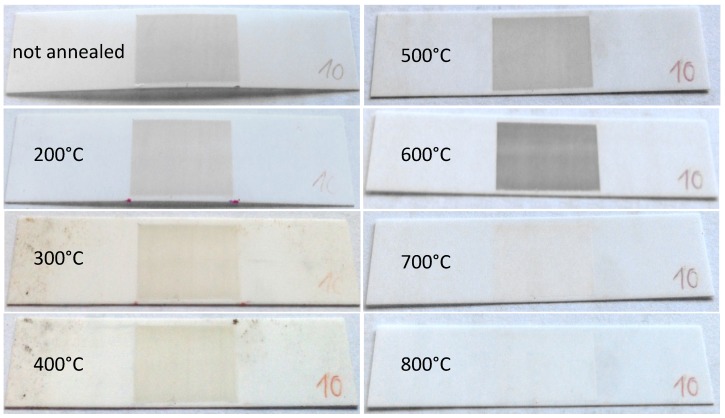
AD Al_2_O_3_ coated alumina substrate annealed in 100 °C steps from 200 °C to 800 °C. Deposition parameters: 100% N_2_, film thickness *t* ≈ 9 µm.

In order to gain information about the stress relaxation in the deposited layers, several samples with different spray parameters and thicknesses between 3 and 10 µm were annealed at 800 °C in air atmosphere. Figure 7 shows one of the coated alumina substrate before annealing and after annealing at different temperatures from 200 up to 800 °C. As deposited, the Al_2_O_3_ layer is clearly visible as a grey film on top of the substrates. With increasing annealing temperature, the color of the deposited layer changed to white/transparent and the stress relaxed completely.

Starting with a stress of 2.1 GPa as deposited, the stress was reduced to 0.9 GPa after annealing at 200 °C. As shown in Figure 7, the stress inside the layer already completely disappeared at 300 °C leading to a flat substrate, whereas the color of the deposited layer first changed from a bright to a deep gray at 600 °C to end up as a white/transparent layer at 800 °C. To clarify this behavior, X-ray diffraction analyses (XRD) were performed at room temperature and while annealing.

**Figure 8:**
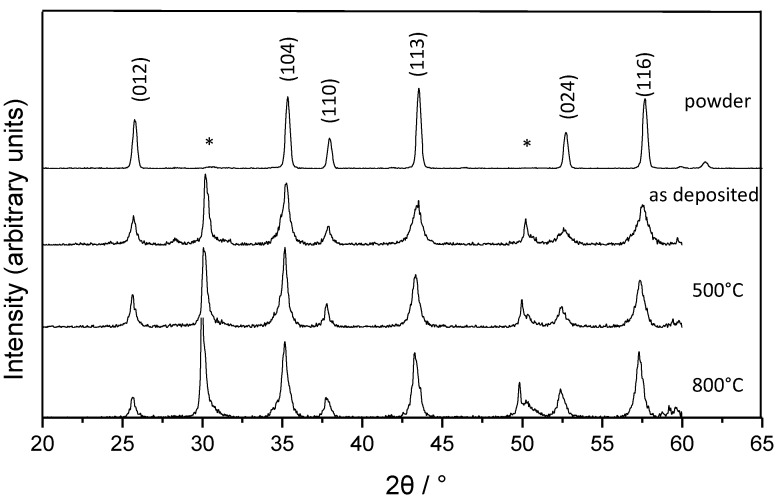
XRD patterns of the raw powder and the AD layer after deposition and while heat treatment at 500 and 800 °C.

The XRD patterns of the raw powder and the deposited film at room temperature and while heat treatments at 500 °C and 800 °C are shown in Figure 8. Peaks related to the PSZ substrate at 2θ = 30° and 50° are marked with a star. All the other peaks belong to alumina and are broadened at room temperature while the width decreases slightly at higher temperatures. This can be explained by the decreased crystallite size after deposition caused by the RTIC mechanism [2] and their growth at higher temperatures. Starting with a crystallite size of 34 nm at room temperature, the crystallites grow to 42 nm at 500 °C and 48 nm at 800 °C. The microstrain decreases from 0.49% at room temperature to 0.33% at 500 °C and 0.28% at 800 °C. So, on the one hand, a part of the reduction of the stress inside the deposited film is connected to crystal growth effects and the release of microstrain of the AD layer which already takes place at temperatures below 500 °C. On the other hand, the total reduction of the stress must be connected to further effects inside the substrate and the deposited film. The density of the deposited films should not affect the measurement as investigations have shown a density up to 99% by SEM cross-section.

One of the additional effects could be the healing of micro cracks [18,19]. Such healing processes are generally based on three different mechanisms. Firstly, chemical reactions may occur. The reaction products alter the nature of the crack. Adhesion, with intermolecular forces being important, and thermal annealing, where diffusion plays the key role [20], are further discussed mechanisms. Chemical reactions can be excluded due to the deposition mechanism and the XRD data. Adhesion through, e.g., van der Waals forces or electrostatic forces are not widely accepted in literature as a mechanism for the healing of micro cracks. However, the process of thermal annealing may explain the observed behavior. Various studies [21,22,23] are showing that the temperature for healing of micro cracks could be significantly below sinter temperature. In case of Al_2_O_3_, this has already been demonstrated for a temperature of 800 °C [22]. Furthermore, Bandyopadhyay *et al.* [21] and Evans *et al.* [23] have shown the dependence of the healing of micro cracks from the length and the width of the cracks. With decreasing crack width and length, the temperature required for micro crack healing decreases.

As a consequence, the micro cracks of the AD layers could be seen in the size of the nm-scaled grain boundaries and could obviously be healed at even lower temperatures. This may explain the release of the stress at such a low temperature of 300 °C.

In addition to this, the change of the color during reduction of the stress inside the deposited film, besides the crystallite growth while annealing, could also be related to the healing of micro cracks. According to Apetz *et al.* [24], the Al_2_O_3_ films should be transparent as they have a grain size below 3 µm. Here, transparency of the coating could only be achieved after annealing at 800 °C. As refraction of light at grain boundaries is the main effect in polycrystalline Al_2_O_3_, micro cracks could have an influence on the refractive behavior of AD layers. Obviously, the color change takes place at 700 °C, whereas the reduction of the film stress already occurs at 300 °C. Therefore, the influence of micro cracks on the optical properties cannot be clarified in this study and further investigations are needed.

## 4. Conclusions

The present study shows that the carrier gas has a significant effect on the formation of stress inside AD layers during deposition. By using oxygen instead of nitrogen or helium as a carrier gas, the stress can be halved. When the substrates are annealed the complete release of the stress occurs below 300 °C. XRD-patterns show crystallite growth from 34 nm to 48 nm and a reduction of the microstrain from 0.49% to 0.28% while annealing the deposited layer at only 800 °C. As the microstrain is not removed completely, other effects like the healing of micro cracks must be responsible for the total reduction of the stress of the substrates at such a low temperature. However, further studies are needed to prove this without a doubt.

## References

[1] Schatt W., Wieters K.P., Kieback B. (2007). Pulvermetallurgie, Technologie und Werkstoffe.

[2] Akedo J. (2008). Room temperature impact consolidation (RTIC) of fine ceramic powder by aerosol deposition method and applications to microdevices. J. Therm. Spray Technol..

[3] Akedo J. (2004). Aerosol deposition method for fabrication of nano crystal ceramic layer. MSF.

[4] Akedo J. (2006). Aerosol deposition of ceramic thick films at room temperature: Densification mechanism of ceramic layers. J. Am. Ceram. Soc..

[5] Sahner K., Kaspar M., Moos R. (2009). Assessment of the novel aerosol deposition method for room temperature preparation of metal oxide gas sensor films. Sens. Actuators B.

[6] Akedo J. (2011). Ceramic coating at room temperature with aerosol deposition method. J. Vac. Soc. Jpn..

[7] Lee D.W., Nam S.M. (2010). Factors affecting surface roughness of Al_2_O_3_ films deposited on Cu substrates by an aerosol deposition method. J. Ceram. Process. Res..

[8] Johnson S.D., Kub F.J., Eddy C.R. (2013). ZnS/Diamond composite coatings for infrared transmission applications formed by the aerosol deposition method. Proc. SPIE.

[9] Ryu J., Kim K.J., Choi J.J., Hahn B.D., Yoon W.H., Lee B.K., Park D.S., Jeong D.Y., Park C. (2009). Flexible dielectric Bi_1.5_Zn_1.0_Nb_1.5_O_7_ thin films on a cu-polyimide foil. J. Am. Ceram. Soc..

[10] Heo Y.J., Kim H.T., Kim K.J., Nahm S., Yoon Y.J., Kim J. (2013). Enhanced heat transfer by room temperature deposition of AlN film on aluminum for a light emitting diode package. Appl. Therm. Eng..

[11] Hirose S., Sakata H., Nakayama C., Koshizuka N., Murakami M., Akedo J. Superconducting MgB_2_ thick films by AD process. Proceedings of the 4th Tsukuba International Coatings Symposium.

[12] Lee D.W., Kim H.J., Kim Y.H., Yun Y.H., Nam S.M. (2011). Growth process of α-Al_2_O_3_ ceramic films on metal substrates fabricated at room temperature by aerosol deposition. J. Am. Ceram. Soc..

[13] Cho S.H., Yoon Y.J. (2013). Multi-layer TiO_2_ films prepared by aerosol deposition method for dye-sensitized solar cells. Thin Solid Films.

[14] Lee B.K., Jung J.H., Hahn B.D., Yoon W.H., Park D.S., Choi J.J., Ryu J., Kim J.W., Ahn C.W., Song K.M. (2011). Dense yttria film deposited on a plasma-sprayed Al_2_O_3_ coating by aerosol deposition. J. Ceram. Sci. Technol..

[15] Akedo J., Lebedev M. (2000). Piezoelectric properties and poling effect of Pb(Zr, Ti)O_3_ thick films prepared for microactuators by aerosol deposition. Appl. Phys. Lett..

[16] Kim H.J., Kim Y.H., Lee J.W., Nam S.M., Yoon Y.J., Kim J.H. (2012). Residual stress relief in Al_2_O_3_-poly-tetra-fluoro-ethylene hybrid thick films for integrated substrates using aerosol deposition. J. Nanoelectron. Optoelectron..

[17] Stoney G. (1909). The tension of metallic films deposited by electrolysis. Proc. R. Soc. Lond. Ser. A Contain. Pap. Math. Phys. Character.

[18] Gupta T.K. (1976). Crack healing and strengthening of thermally shocked alumina. J. Am. Ceram. Soc..

[19] Lange F.F., Gupta T.K. (1970). Crack healing by heat treatment. J. Am. Ceram. Soc..

[20] Case E.D., Smyth J.R., Hunter O., Bradt R.C., Evans A.G., Hasselmann D.P.H., Lange F.F. (1983). Microcrack healing during the temperature cycling off single phase ceramics. Fracture Mechanics of Ceramics.

[21] Bandyopadhyay G., Kennedy C.R. (1977). Isothermal crack healing and strength recovery in U_2 _ subjected to varying degrees of thermal shock. J. Am. Ceram. Soc..

[22] Wiederhorn S.M., Hockey B.J., Roberts D.E. (1973). Effect of temperature on the fracture of sapphire. Philos. Mag..

[23] Evans A.G., Charles E.A. (1977). Strength recovery by diffusive crack healing. Acta Metall..

[24] Apetz R., van Bruggen M.P.B. (2003). Transparent alumina: A light-scattering model. J. Am. Ceram. Soc..

